# Stem Cell Replacement Improves Expression of SMP30 in db/db Mice

**DOI:** 10.3390/ijms161226217

**Published:** 2015-12-16

**Authors:** Ming Li, Kequan Guo, Shigeru Taketani, Yasushi Adachi, Susumu Ikehara

**Affiliations:** 1 Department of Stem Cell Disorders, Kansai Medical University, Hirakata City, Osaka 5731010, Japan; liming@hirakata.kmu.ac.jp; 2 Department of Cardiac Surgery, Beijing Anzhen Hospital affiliated to Capital Medical University, Beijing 100029, China; guokequan@hotmail.com; 3 Department of Microbiology, Kansai Medical University, Hirakata City, Osaka 5731010, Japan; taketans@hirakata.kmu.ac.jp; 4 Division of Clinical Pathology, Toyooka Hospital, Hyogo 6688501, Japan; adachiya250@gmail.com

**Keywords:** SMP30, stem cell replacement, cytokines, fibrosis

## Abstract

We have previously reported that replacing bone marrow stem cells may improve hyperglycemia and oxidative stress in db/db mice, a type 2 diabetic mouse model. Senescence marker protein 30 (SMP30) is an antioxidant protein that decreases with aging. However, it has not been clear whether SMP30 decreases in the livers of obese mice, and whether stem cell replacement would improve SMP30 expression in the liver. Bone marrow stem cells of db/db mice were replaced with the bone marrow stem cells of C57BL/6 mice. Plasma cytokine and insulin levels were measured, and glycogen content, expression of SMP30, and fibrosis in the liver were assessed. Our results showed that stem cell replacement increased the expression of SMP30 in the liver, resulting from decreased plasma inflammation cytokines and hyperinsulinemia in db/db mice. This is the first report that stem cell replacement increased the expression of SMP30 in the liver, and may help prevent fibrosis in the liver of db/db mice.

## 1. Introduction

Senescence marker protein 30 (SMP30) was originally identified as an aging marker protein, the expression of which decreases in an androgen-independent manner in rat liver and kidney cells [1]. SMP30 is a 34 kDa protein, located in the p11.3-q11.2 segment of the X chromosome, and expression of SMP30 decreases in the liver with aging [2]. SMP30 is also known as regucalcin, a calcium-binding protein that plays an important role in cell regulation and signal transduction [3]. Moreover, it has been shown that endogenous regucalcin decreases triglyceride and glycogen levels and regulates glucose metabolism in liver cells of rats, suggesting that it is an important molecule in lipid metabolism, and therefore in diabetes [4]. Regucalcin, as a cytokine, regulates bone homeostasis resulting from suppression of osteoblastogenesis and stimulation of adipogenesis in bone marrow culture *in vitro* [5]. Moreover, regucalcin plays an important role in apoptosis by regulating the expression of apoptosis-related genes [6]. The liver of SMP30-deficient mice was more susceptible to TNFα and Fas-mediated apoptosis when compared with wild type mice. Furthermore, there were large quantities of lipid droplets in the hepatocytes, suggesting that SMP30 is related to abnormal metabolism that may increase the liver’s susceptibility to apoptosis [7]. One report showed that vitamin C levels are related to the expression of SMP30, because SMP30-deficient mice are unable to synthesize vitamin C [8]. SMP30 is a lactone-hydrolyzing enzyme that plays an important role in ascorbic acid biosynthesis [9]. Ascorbic acid is a hexonic sugar that acts as an antioxidant. One report has shown that there was a deficiency of ascorbic acid in SMP30-deficient mice, and did not directly affect lipid oxidation in the liver [10].

Our previous report demonstrated that stem cell replacement improved cytokine imbalances and insulin sensitivity in the obese mouse [11]. However, it was unclear how the expression of SMP30 was improved by stem cell replacement. In this paper, we show that stem cell replacement increases SMP30 expression, with a resultant improvement in metabolic function in the fatty liver of db/db mice.

## 2. Results and Discussion

### 2.1. Ratios in Body Weights and Plasma Cytokine Levels

**Figure 1:**
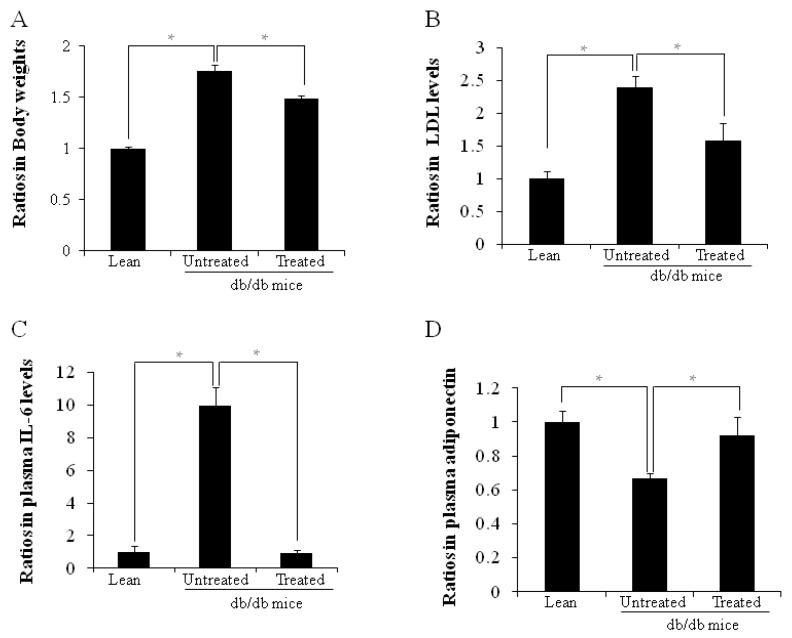
**A**–**D** Ratios of body weight, plasma LDL, IL-6 and adiponectin levels. (**A**) Ratios of body weights are shown (* *p* < 0.05); (**B**) Plasma LDL levels (* *p* < 0.05); (**C**) Plasma IL-6 levels (* *p* < 0.05); (**D**) Plasma adiponectin levels (* *p* < 0.05). The results are mean ± SD, *n* = 6 in each group.

Figure 1A shows the body weight ratios, the body weight ratio in untreated db/db mice being higher than in lean mice (1.76 ± 0.03 *vs.* 1.00 ± 0.02, * *p* < 0.05). The low-density lipoprotein (LDL) level (Figure 1B) was also higher in the untreated db/db mice than the age-matched lean mice (2.40 ± 0.16 *vs.* 1.00 ± 0.11, * *p* < 0.05). Both ratios of body weight and LDL levels were lowered after stem cell replacement, in contrast to untreated db/db mice (* *p* < 0.05). Plasma IL-6 (Figure 1C), and adiponectin (Figure 1D) levels were respectively higher (9.95 ± 1.13 *vs.* 1.00 ± 0.36, * *p* < 0.05) and lower (0.67 ± 0.03 *vs.* 1.00 ± 0.06, * *p* < 0.05) in untreated db/db mice when compared with lean mice. Plasma IL-6 levels decreased after stem cell replacement while adiponectin levels increased (*p* < 0.05).

### 2.2. Morphology of Pancreata, Blood Glucose Levels, and Plasma Insulin Levels

**Figure 2:**
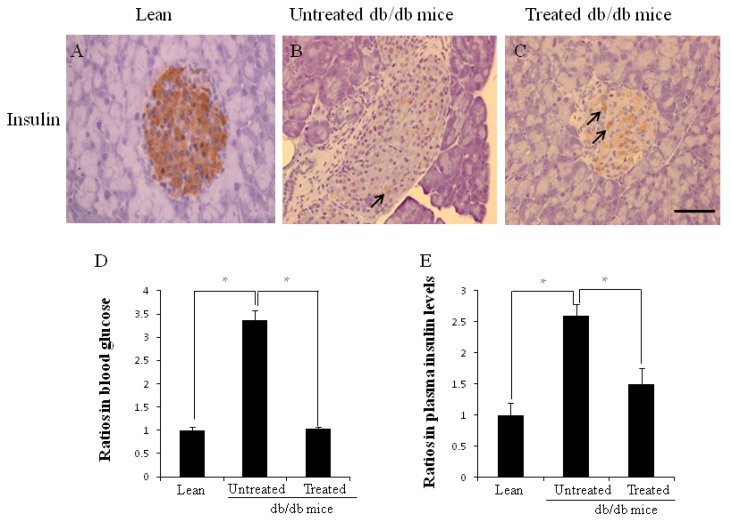
**A**–**E** The morphology of pancreata, ratios of blood glucose levels and plasma insulin levels. Immunochemistry staining for insulin was performed (**A**–**C**); There was considerably more insulin content in residual beta cells (arrows in **C**) when compared to untreated db/db mice (**B**); (**D**) Ratio of blood glucose levels (* *p* < 0.05); (**E**) Ratio of plasma insulin levels (* *p* < 0.05). The results are mean ± SD, *n* = 6 in each group. Scale bar = 25 µm.

There were far fewer insulin-positive cells (brown color, arrow) in the larger islets of untreated db/db mice (Figure 2B) than in those of lean mice (Figure 2A). However, there was increased insulin content in residual beta cells (arrows in Figure 2C) in the treated db/db mice, suggesting that beta cell destruction could be prevented by stem cell replacement. The ratios of fasting blood glucose level (Figure 2D) were higher in the untreated db/db mice than the age-matched lean mice (3.36 ± 0.21 *vs.* 1.00 ± 0.07, * *p* < 0.05). Plasma insulin (Figure 2E) levels were also higher in the untreated db/db mice than the lean mice (2.59 ± 0.19 *vs.* 1.00 ± 0.20, * *p* < 0.05). Both ratios of plasma glucose levels and insulin were decreased significantly after stem cell replacement.

### 2.3. Morphology of Livers and Sirius Red Staining in the Liver 

**Figure 3:**
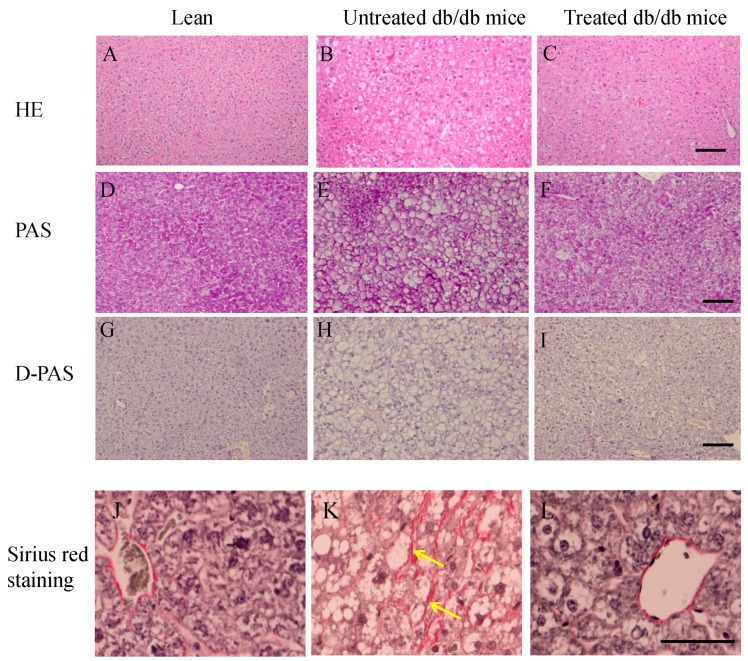
**A**–**L** The morphology of liver. Hematoxylin-eosin staining of livers (**A**–**C**); Glycogen deposits in the hepatocytes (**D**–**F**) by PAS reaction, after diastase digestion (D-PAS) (**G**–**I**); Livers of lean, untreated and treated db/db mice were stained by Sirius red staining. There was more fibrosis (arrows) in the untreated db/db mice (**K**) than lean (**J**) and treated db/db mice (**L**). Scale bar = 25 µm.

Figure 3A–C shows hematoxylin and eosin staining. Enlarged hepatocytes were evident in untreated db/db mice (Figure 3B), while the size of the hepatocytes in the treated db/db mice (Figure 3C) was similar to that of lean mice (Figure 3A). Glycogen deposits, visualized by the periodic acid-Schiff (PAS) reaction, can be seen in the hepatocytes in all groups (Figure 3D–F), but disappear after diastase digestion (Figure 3G–I). However, the glycogen deposit density was lower in the untreated db/db mice than in the lean mice and the treated db/db mice (Figure 3G,I).

Sirius red staining was used to detect fibrosis in the liver (Figure 3J–L). No fibrosis was observed in the lean mice (Figure 3J), but was seen in the untreated db/db mice (arrows in Figure 3K). However, no fibrosis was observed in the treated db/db mice 12 weeks after stem cell replacement (Figure 3L).

### 2.4. Expression of SMP30 and Sirt1 in the Livers

**Figure 4:**
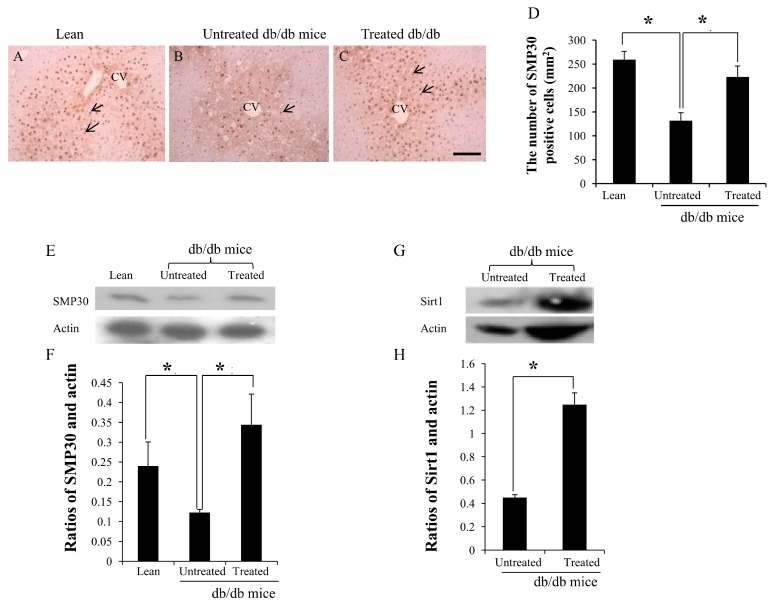
**A**–**D** Immunochemistry staining and results of western blotting for SMP30 and Sirt1 in the livers. Expression of SMP30 decreased more in the liver of untreated db/db mice (arrow in **B**) than lean (arrows in **A**) and treated db/db mice (arrows in **C**); The low SMP30-positive cell counts were improved after stem cell replacement. The expression of SMP30 and Sirt1 was measured by western blotting (**E**,**G**). The ratio of SMP30 and actin, Sirt1, and actin in the treated db/db mice were significantly higher than in the untreated db/db mice (**F**,**H**). CV: central vein. The results are mean ± SD, *n* = 6 in each group. Scale bar = 25 µm. * *p* < 0.05.

SMP-30-positive cells were observed in the liver of lean mice (arrows in Figure 4A) and treated db/db mice (arrows in Figure 4C), but fewer SMP-30-positive cells were observed in the untreated db/db mice (arrows in Figure 4B). The number of SMP30-positive cells per mm^2^ was calculated, the number in the liver of the untreated db/db mice being lower than in the lean mice (132 ± 17/mm^2^ *vs.* 259 ± 15/mm^2^, * *p* < 0.05), while the number in the treated db/db mice was higher than in the untreated db/db mice (223 ± 23/mm^2^ *vs.* 132 ± 17/mm^2^, * *p* < 0.05) (Figure 4D). The expression of SMP30 and actin was detected by western blotting (Figure 4E). Densitometry analyses showed significant increases in the ratios of SMP30 in the treated db/db mice compared to the untreated db/db mice (Figure 4F) (0.34 ± 0.07 *vs.* 0.12 ± 0.01, * *p* < 0.05). Similarly, the expression of Sirt1 and actin was detected by western blotting (Figure 4G). Densitometry analyses showed significant increases in the ratios of Sirt1 in the treated db/db mice than in the untreated db/db mice (Figure 4H) (1.25 ± 0.09 *vs.* 0.45 ± 0.02, * *p* < 0.05).

### 2.5. Discussion

SMP30-positive cells are located mainly around the central veins in the mouse liver, and immunochistochemical analysis has shown the expression decreases with aging [12]. The expression of SMP30 has also been shown to decrease in acute liver injury and tumors in zebrafish [13]. Obesity promotes a low grade inflammatory response, increased secretion of proinflammatory cytokines from immune cells and adipocytes, and decreased adiponectin secretion in the adipocyes. Obesity increases the risk of developing type 2 diabetes mellitus (T2DM) and hepatic steatosis [14]. Db/db mice, a T2DM mouse model, exhibit obesity, hyperglycemia, and hyperinsulinemia. Our previous report showed that stem cell replacement normalized the percentages of lymphocytes in the thymus and also normalized the CD4/CD8 ratio in the peripheral blood, which resulted in decreased plasma IL-6 levels, and increased plasma adiponectin levels. Improved hyperglycemia and fatty liver resulted from improved insulin sensitivity and the expression of Sirt1 and HO-1 in the liver of db/db mice [11,15]. Metformin increases the expression of both Sirt1 and SMP30, improving the functions of endothelial cells. Preventing Sirt1 expression decreased SMP30 expression in cultured endothelial cells, suggesting that SMP30 expression is related to Sirt1 expression [16]. One report has shown that the number of SMP30-positive cells in the normal mouse liver decreases with aging, although there is no significant difference in albumin-positive cells [12]. In this experiment, our results showed that the expression of SMP30 decreased in the liver of db/db mice, and was reversed after stem cell replacement, suggesting that SMP30 may be regulated by Sirt1 expression, which is improved by stem cell replacement. Further studies will focus on the relationship between Sirt1 and SMP30.

In these studies, the body weight, plasma IL-6 levels, and plasma LDL levels were higher in the db/db mice than in the lean mice, and these were improved after stem cell replacement, suggesting that these parameters were related to the expression of SMP30 in the liver. SMP30 has been shown to modulate the balance of protein tyrosine kinase and protein tyrosine phosphates, and also to regulate NF-κB related inflammatory activity [17]. One report showed that, 30 min after glucose administration, blood glucose levels rose higher and insulin levels fell lower in SMP30-deficient mice than in wild-type mice [18]. Another report has shown that SMP30-deficiency induces various organ dysfunctions during the aging process. Hyperlipidemia is a putative contributing factor to nonalcoholic fatty liver disease (NAFLD) development, while abnormal lipid metabolism and lipid accumulation in SMP30-deficient mice indicates that SMP30-deficiency may indirectly lead to NAFLD [19]. Another report showed that hepatic stellate cells were activated by stimulation with insulin and leptin, but inhibited by adiponectin. Moreover, numerous hepatic stellate cells were found to be hypertrophic and to contain abundant microvascular lipid droplets in the cytoplasm in SMP30-deficient mice [20]. Lipid deposition is accompanied by increased oxidative and endoplasmic reticulum stress in SMP30-deficient mice, and SMP30 deficiency contributes to increases in plasma LDL and decreases in high-density lipoprotein (HDL). Moreover, decreased hepatic SMP30 mRNA expression is related to mitochondrial fatty acid oxidation [21].

β-catenin plays a critical role in regulating cell growth and differentiation and apoptosis in the liver pathobiology. SMP30 has been shown to be a target of β-catenin signaling in the liver, and β-catenin regulates vitamin C biosynthesis through SMP30 and l-gulonolactone oxidase expression. β-catenin regulates cell survival through the expression of SMP30 in murine cells. However, in human cells, SMP30 might regulate cell survival by regulation of the redox state of the cell, independent of vitamin C biosynthesis [22]. Oxidative stress is manifested by an imbalance between the formation of pro-oxidants such as reactive oxygen species (ROS) and/or reactive nitrogen species, and the production of antioxidant defenses. Oxidative stress, due to increased production of ROS and decreased antioxidant defense, is observed in both human and experimental models of steatohepatitis [23]. Downregulation of SMP30 is accompanied by an increased generation of the oxygen reactive entity ROS [24]. The relationship between SMP30 expression and oxidative stress is critical for understanding the mechanisms of senescence. Moreover, the activities of inflammatory NF-kB are higher in SMP30-deficient mice, suggesting that SMP30 prevents oxidative stress and chronic inflammation [17]. Indeed, one report indicates that high levels of oxidative stress caused abnormal plasma lipid metabolism and hepatic lipid accumulation in the SMP30 and superoxide dismutase 1-deficient mouse [25]. 

## 3. Experimental Section

### 3.1. Animals

Five-week-old male db/db and lean (BKS.Cg-m+/+Lepr^db^) mice were purchased from Charles River Laboratories (Yokohama, Japan), and male C57BL/6 (B6) mice were purchased from SLC (Shizuoka, Japan). All mice were maintained in animal facilities under specific pathogen-free conditions, and all procedures were performed under protocols approved by the Institutional Animal Care and Use Committee at Kansai Medical University. Each experiment was repeated three times.

### 3.2. Stem Cell Replacement

Bone marrow cells of B6 mice were injected into the recipient (db/db) mice (1 × 10^7^/mouse) by intra bone marrow-bone marrow transplantation as previously described [26], after the recipient had received fractionated irradiation one day before. Simultaneously, the thymi from the newborn B6 mice were engrafted under the renal capsules of the left kidneys of the recipient mice.

### 3.3. Cytokines**,** Low-Density Lipoprotein (LDL) and Insulin Measurements

Plasma adiponectin and IL-6 levels were measured by ELISA assay kits (R&D Systems, Inc., Minneapolis, MN, USA) according to the manufacturer’s protocol. LDL levels were measured according to the manufacturer’s protocol using HDL and LDL/very LDL cholesterol assay kit (Abcam^®^, Cambridge, UK). Plasma insulin levels were measured using an ELISA kit (Morinaga, Yokohama, Japan).

### 3.4. Immunochemistry

The pancreata and livers of the recipients, lean, and db mice were removed three months after the transplantation. The sections (3 µm thick) of paraffin blocks were stained with hematoxylin and eosin after the tissues were fixed. They were then stained with Periodic Acid Schiff including orthoperiodic acid (Wako Pure Chemical Industries, Osaka, Japan) and Schiff’s reagent (Merck, Darmstadt, Germany) after diastase (Wako, Osaka, Japan) digestion to confirm the presence of glycogen deposits. Sirius red staining was used to detect fibrosis in the liver. We used the sirius red stain kit (Polysciences, Inc., Warrington, PA, USA) according to the manufacturer’s instructions. The livers were stained with anti-SMP30 antibody (Cosmo Bio Co., Ltd., Tokyo, Japan). SMP30-positive cells in the liver of each group were calculated using software (Image-pro plus) for Windows. The pancreata were stained with polyclonal guinea pig anti-swine insulin antibody (N1542, Dako Cytomation, Carpinteria, CA, USA). The stained sections were examined on a microscope (Olympus, Tokyo, Japan).

### 3.5. Western Blot Analysis of Sirt1 Expression

At sacrifice, livers were dissected, pooled for each mouse, and used to measure Sirt1. Specimens were stored at −80 °C until assayed. The lysates from the liver tissues were subjected to sodium dodecylsulfate-polyacrylamide gel electrophoresis (SDS-PAGE) and electroblotted onto poly (vinylidene difluoride) (PVDF) membrane (Bio-Rad Laboratories, Hercules, CA, USA). Immunoblotting was carried out with antibodies for SMP30 and Sirt1 (Abcam^®^, Cambridge, UK) and actin (BML, Tokyo, Japan) as the primary antibodies.

### 3.6. Statistical Analysis 

The results are represented as means ± SD. The Student’s *t* test was used to determine any statistical significance. A *p*-value of <0.05 was considered to be a significant difference.

## 4. Conclusion

This is the first report showing that SMP30 expression is decreased in the liver of obese mice, and that stem cell replacement increases its expression, which may in turn attenuate fibrosis in the liver of db/db mice.
